# Identification and Evaluation of Reference Genes for Quantitative PCR Normalization in Alligator Weed Flea Beetle (Coleoptera: Chrysomelidae)

**DOI:** 10.1093/jisesa/ieab067

**Published:** 2021-09-30

**Authors:** Yan-Qiong Guo, Yongchang Yang, Yanping Chai, Ling-Ling Gao, Ruiyan Ma

**Affiliations:** 1 College of Plant Protection, Shanxi Agricultural University, Taigu, Shanxi 030801, China; 2 CSIRO Agriculture and Food, Wembley 6014, Western Australia, Australia

**Keywords:** transcription analysis, reference gene, flea beetle, alligator weed, biological control

## Abstract

Stably expressed reference genes are critical internal standards for the quantification of gene transcription levels using quantitative real-time PCR. Housekeeping genes are commonly used as reference genes but their expressions were variable depending on experimental conditions in many insect species studied. Here we report the identification and evaluation of 10 housekeeping genes in alligator weed flea beetle, *Agasicles hygrophila* Selman & Vogt (Coleoptera: Chrysomelidae), a biocontrol agent of alligator weed. The 10 housekeeping genes are: beta-actin (*Actin*), ribosomal protein L13A (*PRL13a*), succinate dehydrogenase complex subunit A (*SDHA*), ribosomal protein S20 (*RPS20*), ribosomal protein S13 (*RPS13*), glyceraldehyde phosphate dehydrogenase (*GAPDH*), TATA-box-binding protein (*TBP*), ribosomal protein L32 (*RPL32*), tubulin alpha-1 chain (*TUBULIN*), and elongation factor-1 alpha (*ELF*). Five programs, geNorm, NormFinder, BestKeeper, ΔCt method, and RefFinder, were used to evaluate the expression stability of the 10 genes among various *A. hygrophila* body parts and with different nutrient types (starvation, diet types). The expression stability analysis showed that *RPS32* and *RPL13a* were reliable reference genes for the study of gene transcription in different body parts; *Actin* and *RPL13a* were optimal reference genes for different nutrient types. The selections of reference genes were validated using a CarE gene (GeneBank No: KX353552). The results of this study provide useful bases for studies of gene expression in various aspects relating to *A. hygrophila*.

Alligator weed flea beetle, *Agasicles hygrophila* Selman & Vogt, is a well-established biological agent for the control of alligator weed [*Alternanthera philoxeroides* (Mart.) Griseb (Amaranthaceae)] (Spencer and Coulson, 1976; Ma and Wang, 2005). As a successful example of biological control agent, *A. hygrophila* has been studied regarding biology, physiology, ecology, and application security (Maddox 1968, Stewart et al. 1999, Guo et al. 2012). *A. hygrophila* is a monophagous insect feeding exclusively on *A. philoxeroides*. This insect–plant relationship remains unbreakable in any geographical environment. For example, *A. hygrophila* was introduced into China 30 yr ago and its host shift has not been observed so far (Ye et al. 2003, Lu et al. 2010, 2015). Therefore, this unique interaction between *A. hygrophila* and *A. philoxeroides* has lent itself a model system for studying of herbivore-plant specificity.

However, there is little understanding of the underlying mechanism of this strong herbivore-plant specialization. In recent years, studies have been conducted primarily with *A. philoxeroides*, in the aspects of genetic variation (Ma et al. 2013), gene expression in response to abiotic stresses (Jia et al. 2014, 2020a; Guo et al. 2017), as well as olfactory cues in herbivore host shifting (Li et al. 2017). Research of *A. hygrophila* at the molecular level is just emerging (Zhang et al. 2018, 2019; Jia et al. 2020a, b).

Quantitative real-time PCR (RT-qPCR) is the most common tool for gene transcription analysis because of its sensitivity and repeatability (Bustin et al. 2005, Nolan et al. 2006). However, RT-qPCR outcomes are highly dependent on the quality and integrity of the RNAs, the quality and quantity of the template cDNAs, primer specificity, and amplification efficiency. To normalize these variations, internal reference genes are critical for the accurate quantification of the target gene expression. An ideal reference gene should be expressed ubiquitously and insensitive under various experimental conditions.

Most reference genes of RT-qPCR are housekeeping genes because their expressions are ubiquitous and stable regardless of environmental conditions (Pan et al. 2015, Sun et al. 2015). Examples of such genes are NADH oxidase (*NADH*; Li et al. 2013), beta-actin (*Actin*; Zhai et al. 2013), glyceraldehyde-3-phosphate dehydrogenase (*GAPDH*; Zhu et al. 2014), and 18S ribosomal RNA (18S rRNA; Nicot et al. 2005). However, the expression of these popular internal reference genes sometimes varies significantly depending on sample types or experimental conditions (Fu et al. 2013, Li et al. 2013, Zhu et al. 2014). Several studies have indicated that the selection of internal control genes is critical for gene transcription analysis (Arun et al. 2015, Sun et al. 2015) as normalization with unsuitable internal control genes can lead to false results. In fact, fewer genes are stably expressed and suitable for gene expression analysis for all cell and tissue types, or in various experimental conditions (Yang et al. 2014a, Yuan et al. 2014, Zhu et al. 2014). Therefore, the expression profiles of housekeeping genes under various conditions for a given insect species require meticulous evaluation. The stability of housekeeping genes in *A. hygrophila* has not been explored.

To identity suitable reference genes in *A. hygrophila*, 10 candidate reference genes were selected from our in-house transcriptome database. The expression stability of these genes was evaluated in major body parts and nutrient types (starvation, fed with host or non-host plants). Five algorithm-based methods, NormFinder (Andersen et al. 2004), geNorm (Vandesompele et al. 2002), BestKeeper (Pfaffl et al. 2004), the ΔCt method (Nicholas et al. 2006), and RefFinder (Faten et al. 2014) were used to evaluate and rank the stability of these 10 candidate genes for their suitability as reference genes. The results provide much needed guidance for selecting reliable reference genes in gene expression studies in *A. hygrophila*.

## Materials and Methods

### Insects

An *A. hygrophila* colony was established in 2007 from adults collected from *A. philoxeroides* grown at the campus of South China Agricultural University (Wushan, Guangzhou, Guangdong). Since then, the insects had been maintained in a growth chamber (PRX-450C) under the conditions of 26°C, of 85 ± 5% RH, and a photoperiod of 12:12 (L:D) h at the College of Plant Protection, Shanxi Agricultural University. Adult insects (in groups of 10–15 individuals, mixed sexes) were placed in glass jars (7 cm in diameter and 8 cm in height with moist filter paper at the bottom) containing fresh alligator weed plants. The jars were covered with fine muslin cloth fastened with rubber bands. Females laid eggs in clusters on the abaxial surface of leaves. Leaves with eggs were collected and placed in petri dishes (15 cm in diameter) with moist filter paper at the bottom and fresh alligator weed shoots as food source for hatched larvae. The dishes were covered with perforated plastic wrap fastened with rubber bands. When the larvae reached at the third instar, they were transferred to glass jars with fresh alligator weed stems until adults. The alligator weed plants (shoots or stems) were replaced daily.

### Sample Preparation

The samples of *A. hygrophila* body parts: head, midgut, and residue part (body without head and midgut), were collected from 30 adults of mixed sexes (second day after emergence). Four independent biological replicates were prepared. All the samples were flash-frozen in liquid nitrogen and kept at −80°C until RNA extraction.

For nutrient type, 20 or 30 newly emerged adults (<12 h old, male: female = 1:1) of *A. hygrophila* were used as one replication. With the starvation treatment, the 20 adults were placed in a glass jar with only moist filter paper at the bottom; while for the host plant treatment, 20 adults were placed in a glass jar supplied with alligator weed leaves. For the non-host treatment, individual insect was supplied with the leaves of *Beta vulgaris var. cicla*. Thirty adults were prepared to ensure that at least 20 adults had consumed the supplied leaves and could be used for further analysis. All plant materials were replaced twice daily and kept moist with damp filter papers at the bottom of the jar. There were four biological replications (20 beetles each) for each nutrient treatment. After 48 h, the beetles (in groups of 20) were collected for RNA extraction and further analysis.

### Reference Gene Candidates and Primer Design

Ten house-keeping genes including beta-actin (*Actin*), ribosomal protein L13A (*PRL13a*), succinate dehydrogenase complex subunit A (*SDHA*), ribosomal protein S20 (*RPS20*), ribosomal protein S13 (*RPS13*), ribosomal protein L32 (*RPL32*), glyceraldehyde phosphate dehydrogenase (*GAPDH*), TATA-box-binding protein (*TBP*), tubulin alpha-1 chain (*Tubulin*), and elongation factor-1 alpha (*ELF*) were selected from our in-house trancriptome database of *A. hygrophila* previously obtained from beetles in starvation or fed with *B. vulgaris* or alligator weeds, which had a total of 46,151 unigenes (GenBank accession numbers: PRJNA744033). Primers of these 10 genes were designed using the Premier 5 software (http://www.Premierbiosoft.com/primerdesign/index.html). The sequences of the primers used for RT-qPCR are listed in Table 1.

**Table 1.** Primers of the candidate reference genes for RT-qPCR

Gene	Accession number	Primer sequences (5′→3′)	PCR products (bp)	E (%)
*RPS20*	KX271869	F:ACGTTTCGTGTCTGGTTC	110	95.16
		R:TAGTGGTTTTTCGGGATT		
*SDHA*	KX271876	F:CTACAAGATCCCATACCG	112	97.89
		R:CAATCAGAGCCTTTCACT		
*RPS13*	KX271870	F:AGACAGTACAAAATCCCC	126	94.26
		R:CTTCTTCAGCCTCTCAAG		
*RPL32*	KX271871	F:GGATCTATATCCGCTTAGTTTTT	119	100.07
		R:TATCGGTCTGATTGATGTCTG		
*TBP*	KX271877	F:TGGCTATATCTTTTCCTGGTG	121	94.79
		R:ATCCTCGCATTGATGTTTTCT		
*GAPDH*	KX271872	F:TTGGTTATCAACGGACA	199	93.43
		R:ACACATACATAGGGGCG		
*RPL13a*	KX271875	F:CGAGTAGTTGTGCCTGGA	196	92.90
		R:AAGCGTGTTTGGTGATTT		
*TUBLIN*	KX271873	F:CGGAAAATATGAAGGAGA	156	92.14
		R:AAGAGAGAACCGTAGGGA		
*ELF*	KX271874	F:CTCCGTATTCTGAAACCCG	175	93.21
		R:CGCTCAACTGTCCACCCTT		
*ACTIN*	KX271879	F:GGTATGGAATCCTGCGGT	178	99.93
		R:TCTTGATGGTTGATGGGG		

### Total RNA Extraction and cDNA Synthesis

Total RNA was extracted from each insect sample using Trizol reagent (Invitrogen, Carlsbad, CA). To remove potential genomic DNA contamination, the extracts were treated with RNase-free DNase I following the manufacturer’s instructions, and then purified using RNeasy spin columns (Qiagen, Valencia, CA). The RNA was quantified using the NanoVue UV–Vis spectrophotometer (GE Healthcare Bio-Science, Uppsala, Sweden) and examined for its integrity by 1% agarose gel electrophoresis. The first-strand cDNAs were synthesized from 4 µg of total RNA of each sample with an oligo (dT)_18_ primer and M-MLV reverse transcriptase (Fermentas, New York, NY).

### Reverse Transcription Quantitative PCR (RT-qPCR)

RT-qPCR experiments were carried out using the Biosystems 7500 real-time PCR system (Applied Biosystems Inc, Foster, CA) with SYBR Premix Ex Taq TM II kit (Takara, Dalian, China). The cycle parameters consisted of an initial step at 95°C for 10 s, followed by 40 cycles of 95°C for 5 s, 60°C for 31 s, and a final melting curve analysis. The reaction volume was 20 µl containing 10 µl SYBR GREEN Real-time PCR Master mix (TOYOBO), 0.8 µl of each primer (10 mM), 2 µl template (20× diluted), and sterilized water. There were two technical duplicates for each of the four independent biological replications.

### Expression Stability Analysis

The transcription level of each candidate gene was calculated from average Ct value. The expression stability was evaluated with the ΔCt methods (Nicholas et al. 2006), geNorm (Vandesompele et al. 2002), BestKeeper (Pfaffl et al. 2004), NormFinder (Andersen et al. 2004), and RefFinder (Faten et al. 2014) for comprehensive ranking of the tested candidate genes. All evaluations were conducted properly following the instructions of the software.

### Reference Gene Validation

A CarE gene (GeneBank No: KX353552) was used to validate the selected reference genes using the 2^−ΔΔCt^ method. The transcription levels of this gene were estimated using the most stable (NF_1_) and the least stable reference gene (NF_1-2_) and the worst stable (NF_10_) reference gene identified by RefFinder for the samples of different body parts and of different nutrient types. When normalizing using two reference genes, geometric mean was taken as the normalization factor (NF_1-2_) which was calculated from the cycle threshold values of the two reference genes. The results were expressed as the mean ± SE.

The data were statistically analyzed using SPSS software (SPSS, Chicago, IL). One-way ANOVA followed by Turkey’s multiple comparison tests were performed for the effect of reference genes. Statistical difference was claimed when *P* < 0.05.

## Results

### Identification of Reference Gene Candidates

Multiple EST sequences for each selected reference gene candidate were obtained through key word search of the transcriptome dataset previously generated from *A. hygrophila* under the conditions of different nutrient types. The full-length coding sequence of each gene was further blasted against the NCBI database to confirm the gene identity. All 10 candidate genes were submitted to GenBank. The accession numbers and primer sequences used for qRT-PCR are listed in Table 1.

### PCR Amplification Efficiency

Each primer pair of tested genes resulted in a single PCR product as displayed by a single band on the agarose gel or a single peak after melting curve analysis using RT–PCR or RT-qPCR, respectively (Suppl Fig. S1 [online only]). As shown in Table 1, the PCR efficiencies were between 92.14% (*Tubulin*) and 100.07% (*RPL32*) and the coefficients (*R*^2^) were >0.99 for all 10 candidate genes as measured using LinRegPCR program (Table 1).

### Expression Profiles of the Reference Genes Candidates

**Fig. 1.**
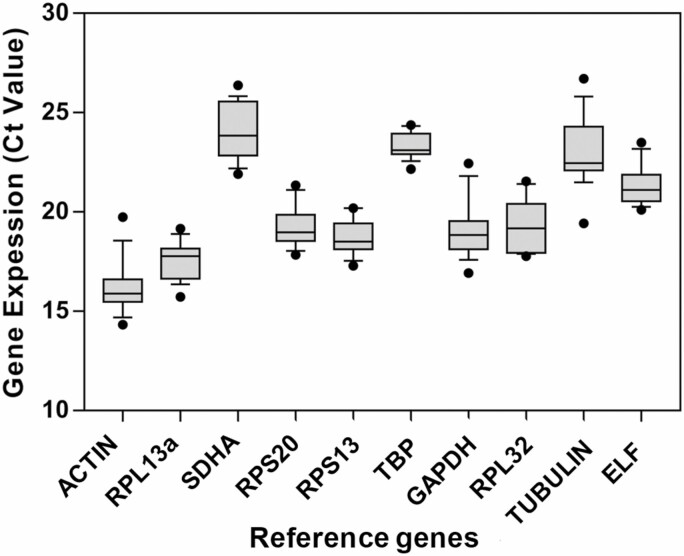
Expression profiles of candidate reference genes of *Agasicles hygrophila*. The expression levels of the genes in 24 tested samples are documented in Ct-value. The dots indicate the maximum or minimum values of the tested samples, while the whiskers indicate the standard error of the means.

The relative abundance and variation of each gene were indicated by the mean and deviation of the Ct values from the 28 samples examined; the lower the Ct value the higher the abundance (Fig. 1). *Actin* was the most abundant (15.89), followed by *RPL13a* (17.77), *RPS13* (18.5), *GAPDH* (18.84), *RPS20* (18.98), *RPL32* (19.18), *ELF* (21.12), *Tubulin* (22.45), *TBP* (23.11), and *SDHA* (23.85). Among the 10 genes, six (*RPL13a*, *RPS20*, *RPS13*, *TBP*, *RPL32*, and *GAPDH*) had lower Ct variations of a similar level with *RPS13* being the lowest. The other four genes (*Actin*, *ELF*, *Tubulin*, and *SDHA*) had relatively higher expression variation with *Actin* being the highest.

### Expression Stability of the Reference Gene Candidates

**Fig. 2.**
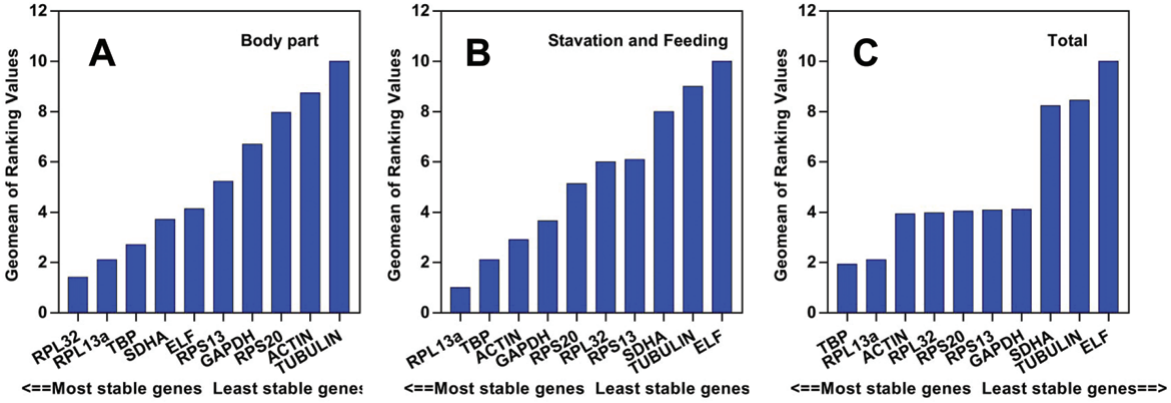
The stability of candidate reference gene expression in different samples evaluated by RefFinder (http://www.leonxie.com/referencegene.php?type=reference). A lower Geomean value indicates higher expression stability.

**Fig. 3.**
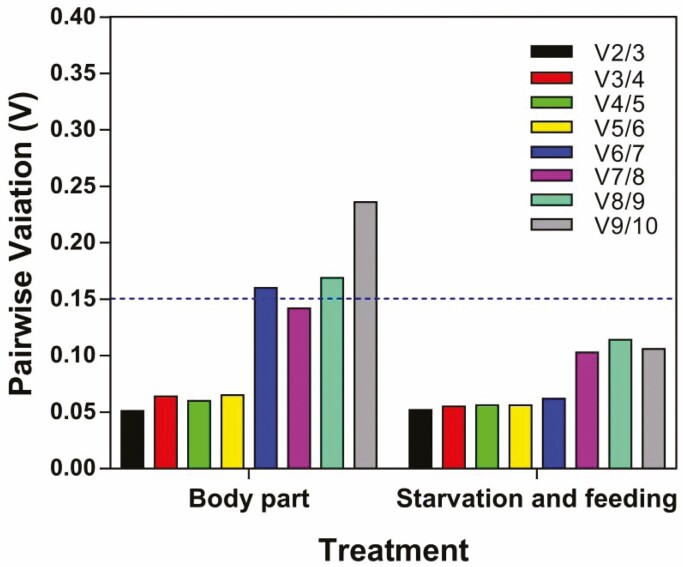
Optimal number of reference genes for different types of *Agasicles hygrophila* sample. Pairwise variation (V) is an index for determining the optimal number of reference genes for accurate RT-qPCR normalization. A cut-off value of 0.15 for pairwise variation was recommended.

Four commonly used statistical programs of geNorm, Normfinder, BestKeeper, ΔCt, and a comprehensive statistical program *RefFinder* were used to evaluate the expression stability of the 10 candidate reference genes in different types of samples. For the samples of different body parts, all programs, except for BestKeeper, identified RPL32 as the most stable gene (Table 2). According to RefFinder, the overall order of these genes from the most stable to the least stable is: *RPL32*, *RPL13a*, *TBP*, *SDHA*, *ELF*, *RPS13*, *GAPDH*, *RPS20*, *Actin*, and *Tubulin* (Fig. 2A). The geNorm analysis revealed that the pair-wise variation value V2/3 was 0.051, which is far less than 0.15, suggesting that two reference genes were enough for accurate normalization of gene expression in body part samples (Fig. 3).

**Table 2.** Stability of reference gene expression under biotic conditions

Conditions	Gene	geNorm stability	Rank	Normfinder stability	Rank	BestKeeper stability	Rank	ΔCt stability	Rank
Body part	*RPL32*	0.21	1	0.11	1	0.73	4	1.01	1
	*RPL13a*	0.21	1	0.19	2	0.77	5	1.05	2
	*TBP*	0.38	3	0.28	3	0.62	2	1.10	3
	*SDHA*	0.46	4	0.45	4	0.63	3	1.14	4
	*ELF*	0.90	7	1.07	6	0.56	1	1.52	7
	RPS13	0.54	5	0.51	5	0.82	6	1.17	5
	GAPDH	0.73	6	1.11	7	1.11	8	1.43	6
	RPS20	1.18	9	1.36	8	0.86	7	1.72	8
	*ACTIN*	1.05	8	1.60	9	1.43	9	1.78	9
	*TUBULIN*	1.44	10	2.39	10	1.73	10	2.51	10
Nutrient types	ACTIN	0.47	1	0.24	1	0.37	1	1.74	1
	*RPL13a*	0.47	1	0.24	2	0.57	5	1.76	2
	*RPS20*	0.50	3	0.24	3	0.49	2	1.81	4
	*TUBULIN*	0.61	4	0.29	5	0.52	3	1.77	3
	*SDHA*	0.82	5	0.90	7	0.54	4	1.92	5
	GAPDH	0.87	6	0.80	6	0.66	6	1.95	6
	*TBP*	1.02	7	0.24	4	0.91	7	2.21	7
	*RPL32*	1.25	8	2.22	8	0.95	8	2.54	8
	*RPS13*	1.41	9	2.44	9	1.04	9	2.70	9
	*ELF*	2.56	10	7.11	10	4.20	10	7.14	10
All conditions	TBP	1.55	7	0.55	1	0.71	1	1.95	2
	RPL13a	1.47	5	0.64	2	0.73	2	1.91	1
	ACTIN	1.40	4	1.06	5	0.90	4	2.02	3
	RPL32	0.90	1	1.60	6	1.17	7	2.21	6
	RPS20	1.52	6	0.90	3	0.80	3	2.04	5
	RPS13	0.90	1	1.70	8	0.96	5	2.24	7
	GAPDH	1.35	3	1.01	4	0.99	6	2.03	4
	SDHA	1.65	8	1.84	9	1.21	8	2.45	8
	TUBULIN	1.77	9	1.64	7	1.34	9	2.47	9
	ELF	2.45	10	5.06	10	2.26	10	5.18	10

For samples of different nutrient types (starvation, fed with host or non-host plant), *Actin* was identified as the most stable gene by geNorm, BestKeeper, and ΔCt (Table 2). The overall ranking (from most stable to least stable) by *RefFinder* is as the following: *Actin*, *RPL13a*, *RPS20*, *Tubulin*, *SDHA*, *GAPDH*, *TBP*, *RPL32*, *RPS13*, and *ELF* (Fig. 2B). This ranking was very different from that of different body parts, suggesting the necessity of selecting different internal reference genes for different tissue types or experimental conditions.

When all sample types were considered, *TBP* and *RPL13a* were the most stable genes identified by Normfinder, BestKeeper, and ΔCt (Table 2). The overall stability ranking by *RefFinder* was as the following: *TBP*>*RPL13a*>*Actin*>*RPL32*>*RPS20*>*RPS13*>*GAPDH*>*SDHA*>*Tubulin*>*ELF* (Fig. 2C). The geNorm analysis revealed that the first V-value < 0.15 appeared at V2/3, suggesting that two reference genes were enough for accurate normalization of all conditions (Fig. 3).

### Validation of Candidate Reference Genes

**Fig. 4.**
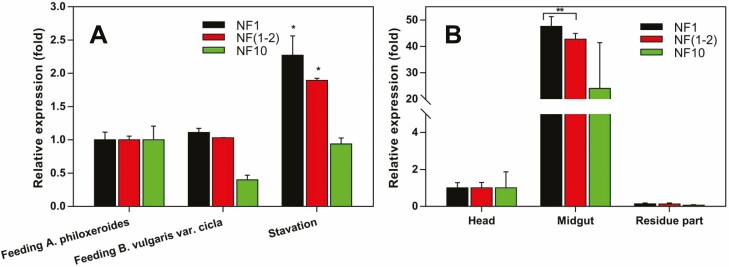
The expression patterns of a CarE gene (Genebank No: KX353552) in different *Agasicles hygrophila* samples for nutrient types (A) or body parts (B) with different internal reference genes. *Statistically significant differences in gene transcript levels among starvation and fed with *A. philoxeroides* (host plant) and *B. vulgaris var. cicla* (non-host plant). **Statistically significant differences in gene transcript levels among samples of different body parts (midgut, head, and residue body part). NF_1_: the most stable reference gene, NF_1-2_: the least stable reference gene, and NF_10_: the worst stable reference gene.

To validate the candidate reference genes, a *CarE* gene (GeneBank No: KX353552) was chosen. According to transcription profiling data collected using the next generation sequencing, this *CarE* gene was upregulated in *A. hygrophila* under starvation condition compared to feeding on the host plant *A. philoxeroides* (Y.-Q.G. et al., unpublished results). When *Actin* or *RPL13a* was used as the reference gene, significantly higher *CarE* transcription level was detected in the starvation insect group than those feeding on the host plants which was consistent with the result from sequencing. However, a different expression pattern was detected when the worst stable gene (*ELF*) was used as a reference that the transcription levels of *CarE* did not differ significantly between insects of starvation and feeding on host plants (Fig. 4A).

The *CarE* gene was expressed at lower levels (<2-folds) in heads and body parts than midguts regardless of the reference gene(s) used. However, for the midgut samples, the transcription level of *CarE* gene was comparable when two stable reference genes *RPL32* or *RPS13a* were used, but was significantly lower when the worst stable gene (*Tubulin*) was used (Fig. 4B).

## Discussion

Reference gene(s) is critical for the normalization of target gene expression using RT-qPCR. In this study, we examined 10 internal candidate reference genes from *A. hygrophila* and evaluated their expression stability with five statistical algorithms. Our results showed that none of the candidate reference genes could serve as a ‘universal’ normalizer. According to RefFinder, which assigns an appropriate weight to an individual gene and gives the overall final ranking, *RPS32* was the most stably expressed gene in samples of different body parts. *RPL13a* appeared to be the best normalization factor for samples of different nutrient types. These results were in line with the reports in *Acyrthosiphon pisum* (Yang et al., 2014a), *Drosophila suzukii* (Zhai *et al.*, 2014), and *Locusta migratoria* (Yang et al., 2014b). It emphasized that the stability of reference gene expression must be verified for different sample typed to ensure a constant level of expression (Thellin et al. 1999). 

*RPL32* (ribosomal protein L32) encodes a ribosomal structural protein that is a component of the 60S subunit (Vorobieva et al. 2008). Previously *RPL32* was shown to be an optimal reference gene to normalize RT-qPCR data in *Kuruma shrimp* (Sellars et al. 2007) and for the analysis of behavioral plasticity in Australian plague locust (Chapuis et al. 2011). In the current study, *RPL32* was ranked at the top in stability for samples of different body parts but ranked lower (as the eighth of the 10 genes) for samples of different nutrient types.

Like *RPL32*, *RPS13*, *RPL13a*, and *RPS20* are all ribosomal proteins (Kenmochi et al. 1998). In this study, *RPL13a* was ranked consistently high in all samples, while by contrast *RPS13* and *RPS20* demonstrated to be less stable among samples of different body parts and nutrient types even though they are also ribosomal proteins. Our results in *A. hygrophila* correlated with those in *Cryptolestes ferrugineus* (Tang *et al.*, 2017) and *Helicoverpa armigera* (Zhang *et al.*, 2015) in which both *RPS20* and *RPS13* appeared to be poorly stable genes under various situations.

*Actin* has been widely used as the ‘universal’ reference gene (even without any validation) as it is considered to be a critical component of the protein scaffold in cytoskeleton maintenance, shape determination, and cell motility. However, our study found that *Actin* displayed very low stability compared to other references particularly in the samples of different body parts (Fig. 2A). Several other studies have also revealed that the expression of *Actin* fluctuated with sample types (e.g., developmental stage, tissue types, etc.; Moshier et al. 1993, Deindl et al. 2002). Furthermore, our results showed that *ELF* and *Tubulin* were poor choices as RT-qPCR reference genes as they were not ranked in the top three reference genes in all cases (Table 2). *GAPDH, RPS20, RPS13*, and *SDHA* were ranked in the middle range in most experimental conditions in this study.

To determine the optimal number of reference genes required for samples of body parts or nutrient types, the pairwise variation (Vn/Vn+1) between two sequential normalization factors (NFn and NFn+1) was calculated by geNorm. A large variation means that an additional reference gene should be abandoned for the calculation of a reliable normalization factor. In this study, we proposed 0.15 as a cut-off value and the V2/3 values were all below 0.15. Therefore, we conclude that two best reference genes were enough for normalizing expression values of target genes under these tested conditions. The validation using the CarE gene provided further information that using two most appropriate genes as references resulted in a more accurate estimation of target gene transcription level than using a single gene. The current study suggests that under most experimental conditions, a single reference gene may not be enough for normalization of gene expression. Two or more reference genes are required to achieve accurate and reliable results (Vandesompele et al. 2002). Our results also demonstrated that the application of the least stable reference gene could result in false interpretation.

### Conclusions

This current study provides a detailed assessment of different candidate reference genes for RT-qPCR studies of *A. hygrophila* with different sample types (body parts and nutrient types). *RPS32* and *RPL13a* were found to be most reliable reference genes for samples of different body parts, while *Actin* and *RPL13a* were optimal reference genes for samples of different nutrient types. This work further demonstrated the importance of reference gene selection and the benefit of combination of at least two reference genes for providing accurate quantification of gene transcription using RT-qPCR. The results of this research provide useful bases for future research in relation to gene transcription in *A. hygrophila*.

## Supplementary Data

Supplementary data are available at *Journal of Insect Science* online.

Fig. S1. The agrose gel profile of the ten candidate reference genes. M, Marker DNA ladder 2000; Templates in the PCR reactions were as follows: 1-ACTIN;2-ELF;3-SDHA;4-TUBULIN;5-TBP;6-GAPDH;7-RPL32;8-RPS20;9-RPL13a;10-RPS13.

Fig. S2. Melting curve of the PCRs for the ten candidate reference genes.

ieab067_suppl_Supplementary_Figures

## References

[CIT0001] Andersen, C. L., J. L. Jensen, and T. F. Ørntoft. 2004. Normalization of real-time quantitative reverse transcription-PCR data: a model-based variance estimation approach to identify genes suited for normalization, applied to bladder and colon cancer data sets. Cancer Res. 64: 5245–5250. 15289330 10.1158/0008-5472.CAN-04-0496

[CIT0002] Arun, A., V. Baumlé, G. Amelot, and C. M. Nieberding. 2015. Selection and validation of reference genes for qRT-PCR expression analysis of candidate genes involved in olfactory communication in the butterfly *Bicyclus anynana*. PLoS One 10: e0120401. 25793735 10.1371/journal.pone.0120401 PMC4368739

[CIT0003] Bustin, S. A., V. Benes, T. Nolan, and M. W. Pfaffl. 2005. Quantitative real-time RT-PCR – a perspective. J. Mol. Endocrinol. 34: 597-601. 15956331 10.1677/jme.1.01755

[CIT0004] Chapuis, M. P., D. Tohidi-Esfahani, T. Dodgson, L. Blondin, F. Ponton, D. Cullen, S. J. Simpson, and G. A. Sword. 2011. Assessment and validation of a suite of reverse transcription-quantitative PCR reference genes for analyses of density-dependent behavioural plasticity in the Australian plague locust. BMC Mol. Biol. 12: 7. 21324174 10.1186/1471-2199-12-7 PMC3048552

[CIT0005] Deindl, E., K. Boengler, N. van Royen, and W. Schaper. 2002. Differential expression of GAPDH and beta3-actin in growing collateral arteries. Mol. Cell. Biochem. 236: 139–146. 12190113 10.1023/a:1016166127465

[CIT0006] Faten, A. T., A. A. Abdel, and B. H. Zhang. 2014. A comprehensive approach to identify reliable reference gene candidates to investigate the link between alcoholism and endocrinology in Sprague-Dawley rats. PLoS One 9: e94311. 24824616 10.1371/journal.pone.0094311 PMC4019588

[CIT0007] Fu, W., W. Xie, Z. Zhang, S. Wang, Q. Wu, Y. Liu, X. Zhou, X. Zhou, and Y. Zhang. 2013. Exploring valid reference genes for quantitative real-time PCR analysis in *Plutella xylostella* (Lepidoptera: Plutellidae). Int. J. Biol. Sci. 9: 792–802. 23983612 10.7150/ijbs.5862 PMC3753443

[CIT0008] Guo, J. Y., J. W. Fu, X. Q. Xian, M. Y. Ma, and F. H. Wan. 2012. Performance of *Agasicles hygrophila* (Coleoptera: Chrysomelidae), a biological control agent of invasive alligator weed, at low non-freezing temperatures. Biol. Invasions 14: 1597–1608.

[CIT0009] Guo, Y. Q., Y. P. Chai, J. Y. Zhang, J. Hao, R. Y. Ma, and L. L. Gao. 2017. The carboxylesterase gene AhCesB1 in alligator weed flea beetle *Agasicles hygrophila*: cloning, sequence analysis and expression profiling. J. Plant Protect. 44: 551–558.

[CIT0010] Jia, D., Y. H. Liu, Z. H. Han, L. L. Zhao, X. Y. Lv, and R. Y. Ma. 2014. Cloning of heat shock protein gene HSP70 in *Agasicles Hygrophila* and its expression in relation to high temperatures. J. Nucl. Agric. Sci. 28: 970–977.

[CIT0011] Jia, D., Z. Y. Ji, X. F. Yuan, B. Zhang, Y. H. Liu, J. Hu, Y. X. Wang, X. C. Li, and R. Y. Ma. 2020a. Molecular cloning and expression profiles of thermosensitive TRP genes in *Agasicles hygrophila*. Insects 11: 531. 10.3390/insects11080531 PMC7570112 32823776

[CIT0012] Jia, D., X. F. Yuan, Y. H. Liu, C. Q. Xu, Y. X. Wang, L. L. Gao, and R. Y. Ma. 2020b. Heat sensitivity of eggs attributes to the reduction in *Agasicles hygrophila* population. Insect Sci. 27: 159–169. 29851277 10.1111/1744-7917.12611

[CIT0013] Kenmochi, N., T. Kawaguchi, S. Rozen, E. Davis, N. Goodman, T. J. Hudson, T. Tanaka, and D. C. Page. 1998. A map of 75 human ribosomal protein genes. Genome Res. 8: 509–523. 9582194 10.1101/gr.8.5.509

[CIT0014] Li, R., W. Xie, S. Wang, Q. Wu, N. Yang, X. Yang, H. Pan, X. Zhou, L. Bai, B. Xu, et al. 2013. Reference gene selection for qRT-PCR analysis in the sweetpotato whitefly, *Bemisia tabaci* (Hemiptera: Aleyrodidae). PLoS One 8: e53006. 23308130 10.1371/journal.pone.0053006 PMC3540095

[CIT0015] Li, N., S. Li, J. Ge, M. Schuman, J. N. Wei, and R. Y. Ma. 2017. Manipulating two olfactory cues causes a biological control beetle to shift to non-target plant species. J. Ecol. 105: 1534–1546.

[CIT0016] Lu, J., L. Zhao, R. Ma, P. Zhang, R. Fan, and J. Zhang. 2010. Performance of the biological control agent flea beetle *Agasicles hygrophila* (Coleoptera: Chrysomelidae), on two plant species *Alternanthera philoxeroides* (alligatorweed) and *A. sessilis* (joyweed). Biol. Control 54: 9–13.

[CIT0017] Lu, J., L. Zhao, N. Li, D. Jia, Y. Guo, J. Wei, R. Fan, and R. Ma. 2015. Performance of the alligatorweed flea beetle, *Agasicles hygrophila*, on nontarget plant species. J. Aquat. Plant Manage. 53: 88–94.

[CIT0018] Ma, R., and R. Wang. 2005. Invasive mechanism and biological control of alligatorweed, *Alternanthera Philoxeroides* (Amaranthaceae), in China. Chin. J. Appl. Environ. Biol. 11: 246-250.

[CIT0019] Ma, R. Y., X. Y. Jia, W. Z. Liu, R. H. Laushman, L. L. Zhao, D. Jia, and R. Wang. 2013. Sequential loss of genetic variation in flea beetle *Agasicles hygrophila* (Coleoptera: Chrysomelidae) following introduction into China. Insect Sci. 20: 655–661. 23956178 10.1111/1744-7917.12025

[CIT0020] Maddox, D. M. 1968. Bionomics of an alligatorweed flea beetle, *Agasicles* sp. in Argentina. Ann. Entomol. Soc. Am. 61: 1299–1305.

[CIT0021] Moshier, J. A., T. Cornell, and A. P. Majumdar. 1993. Expression of protease genes in the gastric mucosa during aging. Exp. Gerontol. 28: 249–258. 8344396 10.1016/0531-5565(93)90032-9

[CIT0022] Nicholas Silver S. B., J. Jiang, and S. L. Thein. 2006. Selection of housekeeping genes for gene expression studies in human reticulocytes using realtime PCR. BMC Mol. Biol. 7: 33. 17026756 10.1186/1471-2199-7-33 PMC1609175

[CIT0023] Nicot, N., J. F. Hausman, L. Hoffmann, and D. Evers. 2005. Housekeeping gene selection for real-time RT-PCR normalization in potato during biotic and abiotic stress. J. Exp. Bot. 56: 2907–2914. 16188960 10.1093/jxb/eri285

[CIT0024] Nolan, T., R. E. Hands, and S. A. Bustin. 2006. Quantification of mRNA using real-time RT-PCR. Nat. Protoc. 1: 1559–1582. 17406449 10.1038/nprot.2006.236

[CIT0025] Pan, H., X. Yang, K. Bidne, R. L. Hellmich, B. D. Siegfried, and X. Zhou. 2015. Selection of reference genes for RT-qPCR analysis in the monarch butterfly, *Danaus plexippus* (L.), a migrating bio-indicator. PLoS One 10: e0129482. 26030778 10.1371/journal.pone.0129482 PMC4452232

[CIT0026] Pfaffl, M. W., A. Tichopad, C. Prgomet, and T. P. Neuvians. 2004. Determination of stable housekeeping genes, differentially regulated target genes and sample integrity: BestKeeper-Excel-based tool using pair-wise correlations. Biotechnol. Lett. 26: 509–515. 15127793 10.1023/b:bile.0000019559.84305.47

[CIT0027] Sellars, M. J., T. Vuocolo, L. A. Leeton, G. J. Coman, B. M. Degnan, and N. P. Preston. 2007. Real-time RT-PCR quantification of Kuruma shrimp transcripts: a comparison of relative and absolute quantification procedures. J. Biotechnol. 129: 391–399. 17350129 10.1016/j.jbiotec.2007.01.029

[CIT0028] Spencer, N. R., and J. R. Coulson. 1976. The biological control of alligatorweed, *Alternanthera philoxeroides*, in the United States of America. Aquat. Bot. 2: 177–190.

[CIT0029] Stewart, C. A., R. B. Chapman, R. M. Emberson, P. Syrett, and C. M. A. Frampton. 1999. The effect of temperature on the development and survival of *Agasicles hygrophila* Selman & Vogt (Coleoptera: Chrysomelidae), a biological control agent for alligator weed (*Alternanthera philoxeroides*). New Zeal. J. Zool. 26: 11–20.

[CIT0030] Sun, M., M. X. Lu, X. T. Tang, and Y. Z. Du. 2015. Exploring valid reference genes for quantitative real-time PCR analysis in *Sesamia inferens* (Lepidoptera: Noctuidae). PLoS One 10: e0115979. 25585250 10.1371/journal.pone.0115979 PMC4293147

[CIT0031] Tang, P. A., J. Y. Duan, H. J. Wu, X. R. Ju, and M. L. Yuan. 2017. Reference gene selection to determine differences in mitochondrial gene expressions in phosphine-susceptible and phosphine-resistant strains of *Cryptolestes ferrugineus*, using qRT-PCR. Sci. Rep. 7: 7047. 28765619 10.1038/s41598-017-07430-2 PMC5539111

[CIT0032] Thellin, O., W. Zorzi, B. Lakaye, B. De Borman, B. Coumans, G. Hennen, T. Grisar, A. Igout, and E. Heinen. 1999. Housekeeping genes as internal standards: use and limits. J. Biotechnol. 75: 291–295. 10617337 10.1016/s0168-1656(99)00163-7

[CIT0033] Vandesompele, J., K. D. Preter, F. Pattyn, B. Poppe, N. V. Roy, A. D. Paepe, and F. Speleman. 2002. Accurate normalization of real-time quantitative RT-PCR data by geometric averaging of multiple internal control genes. Genome Biol. 3: 00341–003411. 10.1186/gb-2002-3-7-research0034 PMC126239 12184808

[CIT0034] Vorobieva, N. V., M. L. Filipenko, G. G. Karpova, N. P. Mertvetsov, and A. S. Graphodatsky. 2008. Assignment of the L32 ribosomal protein gene (RPL32) to human chromosome 3q13.3->q21 by in situ hybridization. Cytogenet. Genome Res. 77: 190–191. 10.1159/000134573 9284913

[CIT0035] Yang, C., H. Pan, Y. Liu, and X. Zhou. 2014a. Selection of reference genes for expression analysis using quantitative real-time PCR in the pea aphid, *Acyrthosiphon pisum* (Harris) (Hemiptera, Aphidiae). PLoS One 9: e110454. 25423476 10.1371/journal.pone.0110454 PMC4244036

[CIT0036] Yang, Q., Z. Li, J. Cao, S. Zhang, H. Zhang, X. Wu, Q. Zhang, and X. Liu. 2014b. Selection and assessment of reference genes for quantitative PCR normalization in migratory locust *Locusta migratoria* (Orthoptera: Acrididae). PLoS One 9: e98164. 24887329 10.1371/journal.pone.0098164 PMC4041718

[CIT0037] Ye, W., J. Li, H. Cao, and X. Ge. 2003. Genetic uniformity of *Alternanthera Philoxeroides* in south China. Weed Res. 43: 297–302.

[CIT0038] Yuan, M., Y. Lu, X. Zhu, H. Wan, M. Shakeel, S. Zhan, B. R. Jin, and J. Li. 2014. Selection and evaluation of potential reference genes for gene expression analysis in the brown planthopper, *Nilaparvata lugens* (Hemiptera: Delphacidae) using reverse-transcription quantitative PCR. PLoS One 9: e86503. 24466124 10.1371/journal.pone.0086503 PMC3900570

[CIT0039] Zhai, Y., J. Zhang, Z. Sun, X. Dong, Y. He, K. Kang, Z. Liu, and W. Zhang. 2013. Proteomic and transcriptomic analyses of fecundity in the brown planthopper *Nilaparvata lugens* (Stal). J. Proteome Res. 12: 5199–5212. 24083549 10.1021/pr400561c

[CIT0040] Zhai, Y., Q. Lin, X. Zhou, X. Zhang, T. Liu, and Y. Yu. 2014. Identification and validation of reference genes for quantitative real-time PCR in *Drosophila suzukii* (Diptera: Drosophilidae). PLoS One 9: e106800. 25198611 10.1371/journal.pone.0106800 PMC4157791

[CIT0041] Zhang, S., S. An, Z. Li, F. Wu, Q. Yang, Y. Liu, J. Cao, H. Zhang, Q. Zhang, and X. Liu. 2015. Identification and validation of reference genes for normalization of gene expression analysis using qRT-PCR in *Helicoverpa armigera* (Lepidoptera: Noctuidae). Gene 555: 393–402. 25447918 10.1016/j.gene.2014.11.038

[CIT0042] Zhang, H., M. Zhao, Y. Liu, Z. Zhou, and J. Guo. 2018. Identification of cytochrome P450 monooxygenase genes and their expression in response to high temperature in the alligatorweed flea beetle *Agasicles hygrophila* (Coleoptera: Chrysomelidae). Sci. Rep. 8: 17847. 30552348 10.1038/s41598-018-35993-1 PMC6294762

[CIT0043] Zhang, H., Y. Wang, Y. Liu, M. Zhao, J. Jin, Z. Zhou, and J. Guo. 2019. Identification and expression patterns of three vitellogenin genes and their roles in reproduction of the alligatorweed flea beetle *Agasicles hygrophila* (Coleoptera: Chrysomelidae). Front. Physiol. 10: 368. 31001144 10.3389/fphys.2019.00368 PMC6454870

[CIT0044] Zhu, X., M. Yuan, M. Shakeel, Y. Zhang, S. Wang, X. Wang, S. Zhan, T. Kang, and J. Li. 2014. Selection and evaluation of reference genes for expression analysis using qRT-PCR in the beet armyworm *Spodoptera exigua* (Hübner) (Lepidoptera: Noctuidae). PLoS One 9: e84730. 24454743 10.1371/journal.pone.0084730 PMC3893131

