# Supplementary material for: Identification and Evaluation of Reference Genes for Quantitative PCR Normalization in Alligator Weed Flea Beetle (Coleoptera: Chrysomelidae)
Source: J Insect Sci. 2021 Sep 30;21(5):9. doi: 10.1093/jisesa/ieab067 (PMC8482964; doi:10.1093/jisesa/ieab067)
Supplement: ieab067_suppl_Supplementary_Figures [file ieab067_suppl_supplementary_figures.pptx]

## Slide 1
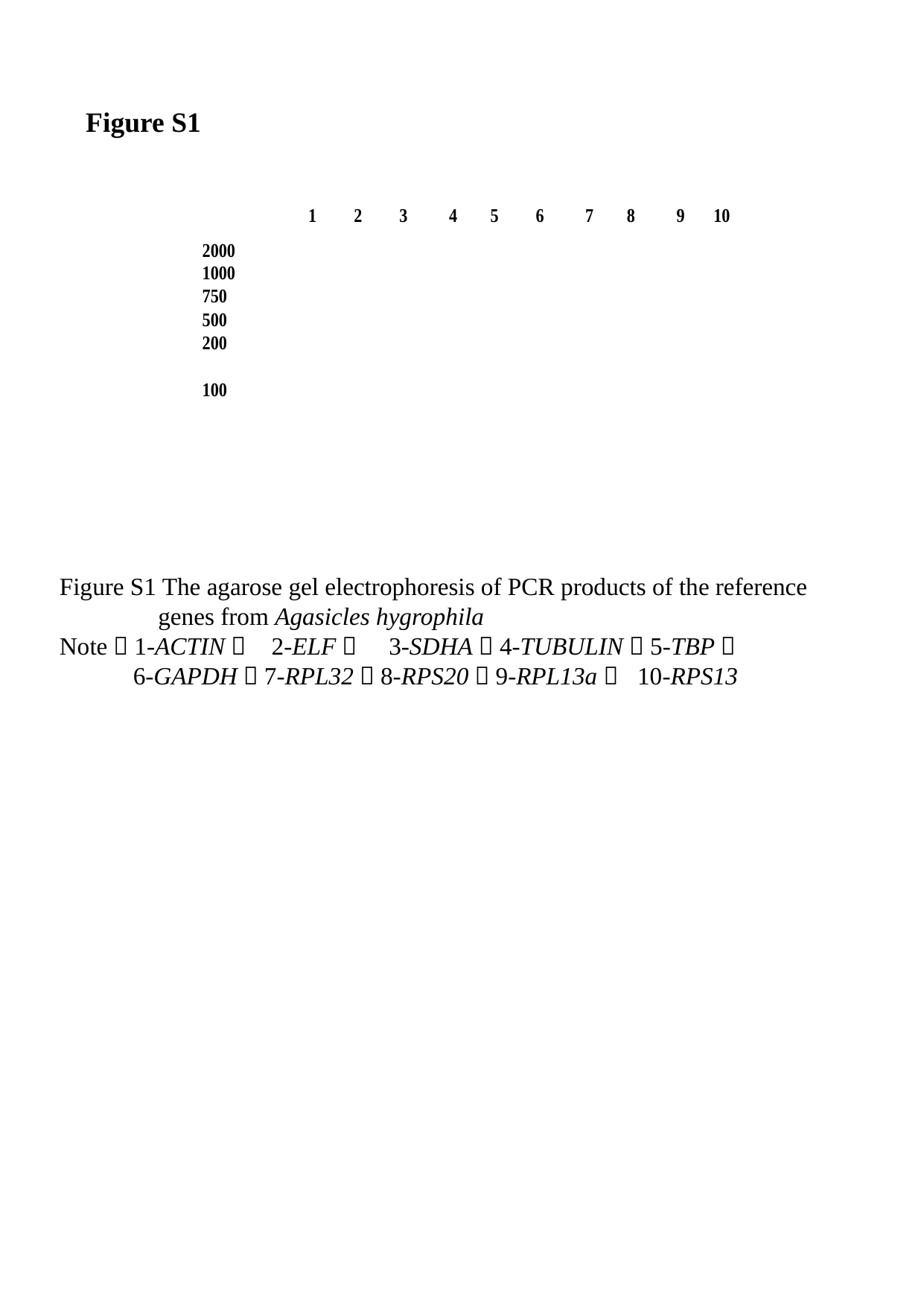

Figure S1
Figure S1 The agarose gel electrophoresis of PCR products of the reference genes from Agasicles hygrophila
Note：1-ACTIN； 2-ELF； 3-SDHA；4-TUBULIN；5-TBP；
 6-GAPDH；7-RPL32；8-RPS20；9-RPL13a； 10-RPS13

## Slide 2
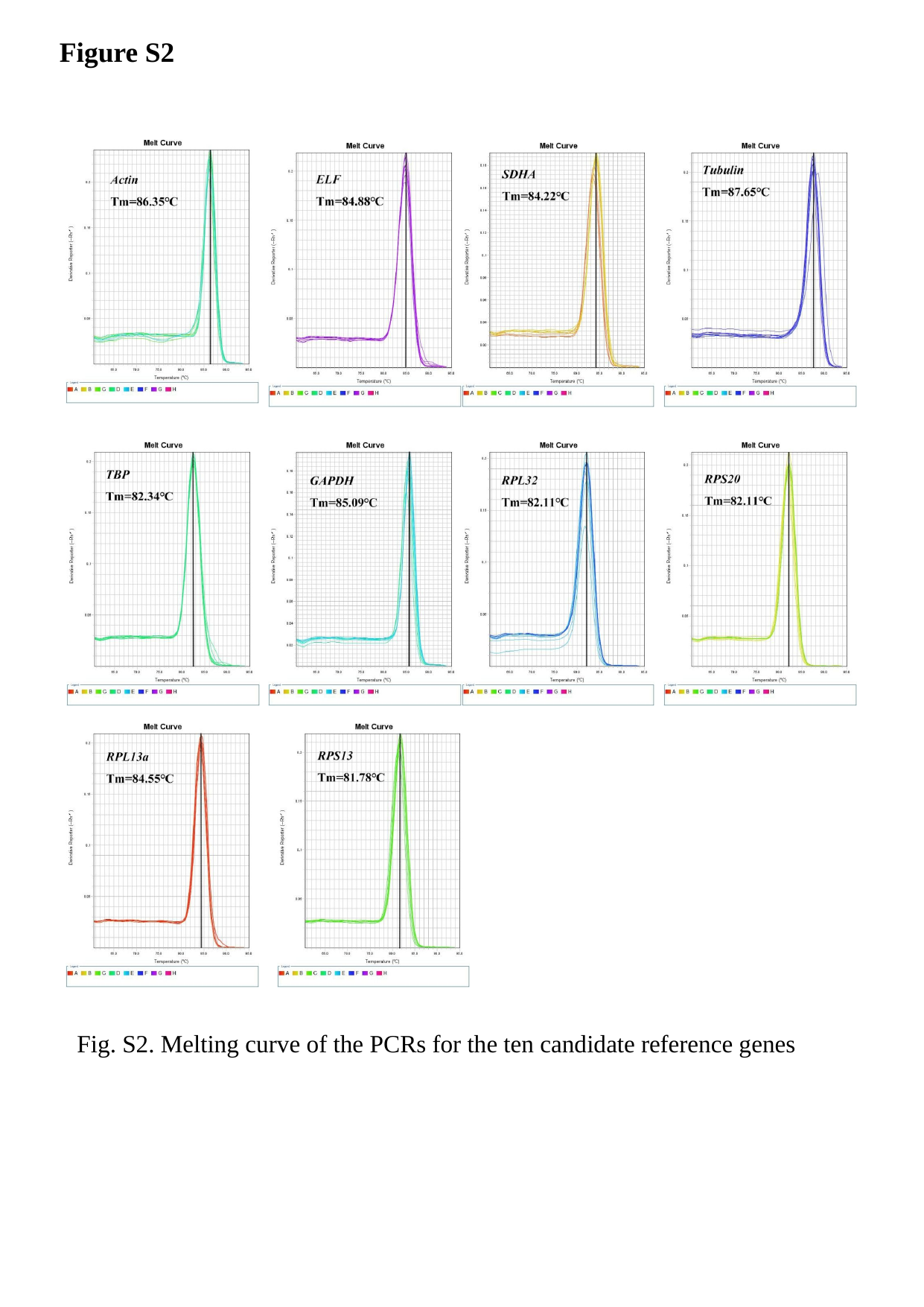

Figure S2
Fig. S2. Melting curve of the PCRs for the ten candidate reference genes
